# Eczema: etiology, subtypes, therapeutic approaches and socioeconomic impact

**DOI:** 10.3389/falgy.2025.1675475

**Published:** 2026-01-12

**Authors:** Marizé Cuyler, Danielle Twilley, Namrita Lall

**Affiliations:** 1 Department of Plant and Soil Sciences, University of Pretoria, Pretoria, Gauteng, South Africa; 2 School of Natural Resources, University of Missouri, Columbia, MO, United States; 3 College of Pharmacy, JSS Academy of Higher Education and Research, Bonne Terre, Vacoas-Phoenix, Mauritius

**Keywords:** alternative treatments, conventional treatments, eczema, etiology, socioeconomic impact

## Abstract

Eczema is an inflammatory skin condition that affects individuals of all ages worldwide. Patients may develop various forms of eczema, including atopic dermatitis, contact dermatitis, which is often associated with an allergic response to various stimuli, dyshidrotic eczema which develops on the palms and soles, asteatotic dermatitis that predominantly occurs in elderly patients, nummular eczema characterized by its cylindrical shape lesions and seborrheic dermatitis often located on patient's scalps, back, face and chest. Extensive studies have been conducted on atopic dermatitis, however, limited information such as their etiology, effect on the immune system and potential treatments are available on the other types of eczema. The socioeconomic impacts of eczema include the cost of conventional treatments such as corticosteroids, immunosuppressive agents and phototherapy, expenses related to specialists’ consultation and the effect on work and school productivity. The impact of atopic dermatitis on patients’ quality of life, social functioning and individual healthcare expenses has been extensively studied in other countries but remains underreported in South Africa. Reports have estimated the annual direct and indirect costs in Australia, the United Kingdom and the United States, however reports are limited for South Africa. This study aimed to provide information on the different types of eczema's etiology, their respective socioeconomic impact in South Africa in correlation to the above mentioned inflated yearly cost, and conventional, targeted and alternative treatments commercially available. Several knowledge gaps were identified in this study, including the limited availability of information on asteatotic dermatitis, dyshidrotic dermatitis and nummular eczema, the effect most commercially available treatments have on other eczema subtypes and an in-depth review of the socioeconomic impact of eczema within the African continent.

## 1 Introduction

Eczema is a collective term for noncontagious inflammatory skin conditions which can be atopic dermatitis (most common form of eczema) but also includes various other forms of eczema such as contact dermatitis, dyshidrotic eczema, asteatotic dermatitis, nummular eczema and seborrheic dermatitis (1, 2). These types of eczema may be presented as an acute or chronic form, can recur and in some cases, a patient can develop more than one type of eczema (2). The various types are often misdiagnosed as symptoms associated with each type and where they are located on the patient often overlap due to limited information regarding the etiology and treatment on a few of the eczema types. Of the various types, atopic dermatitis is the most common and has previously been extensively researched. Statistics on eczema in first world countries such as the United States and Europe is widely available, however, the prevalence of the condition in third world countries such as Africa are not well reported. In 2021, Hadi et al. published a review article which evaluated the prevalence of eczema across the African continent. Data on individuals aged 4–30 years were available from six countries: Tunisia, Rwanda, Namibia, Gabon, Ghana, and Senegal (3–7).

Extensive research into the socioeconomic impact of eczema on various countries including America and parts of Europe have been conducted, however, the costs are often reported in dollars (8). One of the countries mentioned in the study was South Africa, therefore this study aimed to provide estimated values based on recent currency exchange rates and inflation to illustrate the socioeconomic impact of eczema on South Africa. The study further aimed to collate information on the different types of eczema in respect to their etiologies and included current conventional treatments such as corticosteroids, phototherapy and immunosuppressors, alternative treatments such as phytomedicine consisting of Traditional Chinese Medicine (TCM) and natural Western treatments, and targeted treatments such as biologics and small molecules.

## 2 Materials and methods

A structured literature review was conducted using various scientific databases including Google Scholar, PubMed, National Institute of Health (NIH), Wiley, Springer and Elsevier. Scientific publications and book chapters were selected that were published between the year 1972–2024. Keywords included “types of eczema”, “socioeconomic impact”, “corticosteroids”, “natural eczema treatments”, “secondary metabolites used against eczema”, “targeted treatment”, “neuroinflammation”, “endocannabinoids”, “biologics”, “small molecules”, “cannabinoid orientated treatments” and “conventional treatments”, while some were identified using targeted searches on specific topics such as “contact dermatitis”, “dyshidrotic dermatitis”, “physical, biochemical and immune barrier”, “asteatotic dermatitis”, “seborrheic dermatitis”, “UV treatment”, “azathioprine”, “mycophenolate”, “dupilumab”, “nemolizumab”, “delgocitinib”, “ruxolitinib”, “gusacitinib”, “upadacitinib” and “nummular eczema”.

A total of over 400 scientific publications were reviewed to collect information on the different types of eczema, their potential etiology, the socioeconomic impact and various treatments currently available. The potential etiology, location and pathogenesis of the different types of eczema were summarized in Table 1. Limited information on the socioeconomic impact of eczema in Africa could be found, thus the literature search was not limited to Africa and included studies conducted globally. In Section 4, the equivalent costs related to the socioeconomic impact were adjusted for inflation using data from https://www.inflationtool.com. As mentioned in Section 5, the estimated cost in South Africa was calculated based on the exchange rate as of April 22, 2025. In Sections 6 and 7, chemical structure of potentially active secondary metabolites and corticosteroid actives were drawn using BIOVIA 2017 software, while the accepted species name in Section 7 was validated using https://wfoplantlist.org/.

**Table 1:** An overview comparing the various types of eczema in relation to their distribution on the body, frequency of occurrence, and contributing etiological factors.

Type of eczema	Location	Prevalence	Symptoms associated with the condition	Potential causation	Pathogenesis of the condition	References
Atopic dermatitis	Elbows, knees, cheeks, forehead and outer areas of children's limbs younger than 4 years.	10%–20% in children and 3%–5% in adults	Intolerable itching sensation, erythema and lichenification	Inside-out hypothesis • Immunological defects, which may cause sensitivity to immunoglobulin E (IgE). Outside-in hypothesis • Skin barrier disruption due to environmental change and genetic mutation.	Acute: T-helper (Th)-2 and Th-22 lymphocyte imbalance leading to interleukin (IL)-4, IL-5, tumor necrosis factor alpha (TNF-α) and IL-13 expression promoting IgE antibodies. Chronic: Th-1 lymphocyte imbalance leading to the expression of interferon-gamma (IFN-γ), IL-8, TNF-α and IL-12, which suppress Th-2 reducing the expression of IgE antibodies to normal levels. Reduction of beta-glucocerebrosidase (β-GlcCer’ase), acidic sphingomyelinase (aSMase) and phospholipase A2 (sPLA2) and the upregulation of serine proteases. The main genetic mutation is the loss-of-function mutation in the filaggrin gene (*FIL*).	(1, 2, 12, 13, 32, 34–37, 39–41, 170)
Contact dermatitis	Any part of the body that has been exposed to the allergen.	Third most common skin disease in Europe.	Oedema, erythema and intense pruritus. Patients with chronic contact dermatitis can experience symptoms of hyperkeratosis, lichens and cracked skin.	Irritant contact dermatitis (ICD) • Most common form caused by chemical or physical injuries Allergic contact dermatitis (ACD) • Exposure to hapten or small molecules such as nickel, fruits and perfumes. Protein contact dermatitis (PCD) • Proteins that have a molecular weight of >10,000 daltons and are unrelated to haptens.	Allergens enter the skin, which are processed and presented to T and B cells. This converts native T cells to effector T cells and allows B cells to release IgE antibodies. • When re-exposed to the allergen, the effector T cells recognize the antigen and synthesis pro-inflammatory cytokines including TNF-α and IL-1β initiating an inflammatory response while IL-4 promote B cell activation increasing the production of IgE antibodies.	(2, 42–44, 171)
Dyshidrotic eczema	Palms, soles and lateral sections of the fingers specifically where there is a high density of sweat glands.	5%–20% of eczema cases that develop on the hand	Small itchy blisters that persist for 2–3 weeks at reoccurring intervals. When it erupted, vesicular development occurs further aggravating the itching sensation.	Hypothesized to have a similar causation as contact dermatitis due to patient's sensitivity towards various metals, fungal infections, photoinduction and intravenous immunoglobulin therapy.	Hypothesized to have similar pathogenesis as contact dermatitis	(2, 48, 50)
Asteatotic dermatitis	Throughout the body	Around 70% of those over the age of 60 years.	Dry, rough and scaling skin that is itchy and inflamed	Increases transepidermal water loss (TEWL) due to the lack of fatty acids/lipids within the epidermis. Itching sensation is potentially due to C-fibers that transfer the sensation to the cerebral cortex where it is processed.	The lack of lipids prevents the entrapment of moisture within the stratum corneum increasing TEWL. Moreover, abnormal unmyelinated nerve fibers or C-fibers increase the transfer of the itching stimulation to the cerebral cortex promoting the sensation.	(10, 11, 52, 172)
Nummular eczema	Limbs and trunk	Rare type of eczema that is present in patients between 18 and 64 years of age.	Small round patches that have a thin circumference and are often itchy	The condition has been associated with environmental factors, allergens, and nutritional factors.	Speculation that the condition may be due to the overexpression of IL-1, IL-2 and IL-6 as these indirectly affect the production of IL-10, IL-12 and IL-17.	(54–56, 58–60)
Seborrheic dermatitis	Scalp, face, chest, back and groins where large density of sebaceous glands are located	Varies between age groups with a high occurrence in infants, adolescent and young adults then a notable rise in patients older than 50.	Lesions located in the mentioned areas that are often greasy	Factors that have been associated with the cause of the condition include hormone levels, comorbid conditions and cytokines that induce immunological effects. Most studies associate the high presence of *Malassezia* fungal species as the potential cause.	IgG antibodies that are stimulated by IL-4, IL-13 and IL-10.	(61–66, 173)

## 3 Structural and immunological features of eczema skin

Eczema affects the physical, biochemical and immune barriers of the skin. The physical barrier consists of the stratum corneum and tight junctions, which prevent the penetration of chemicals and microbes into deeper layers of the skin and can withstand mechanical damage. The physical barrier is comprised of keratinocytes and structural proteins such as keratin, filaggrin and loricrin, which maintain the integrity of the skin barrier. Deficiencies in these proteins have been shown to disrupt the skin barrier increasing the permeability which compromises the deeper layers of the skin (9).

Lipids, including ceramides located in the stratum corneum, are predominantly produced by keratinocytes through a process known as keratinization, which traps moisture and forms multi-lamellar structures between the stratum corneum cells. These cells require a water concentration of 10%–20% to maintain their integrity, however, moisture entrapment decreases significantly when ceramide production is reduced causing the skin to split and fissure (10, 11). Under normal conditions, keratinization occurs at a skin pH of 4.7–5.75 as this pH contributes to the homeostasis of the barrier and ensures the stratum corneum remains intact. Eczema patients typically exhibit an elevated skin pH of 6, causing a disruption to the keratinization process and the upregulation of serine proteases. These serine proteases downregulate the secretion of lamellar bodies within the lamellar structure reducing the bacterial flora on the skin, affecting the biochemical barrier (12, 13).

The biochemical barrier contains lipids, antimicrobial peptides, including cathelicidins, beta defensins (β-defensins), psoriasin, lactoferrin and acids that act as an innate immune barrier to prevent microorganisms and their proteins from entering. Upon disruption of the physical barrier, antimicrobial proteins are expressed to prevent the onset of infection and the spread of pathogens present on the skin (9, 14). Studies have shown reduced levels of cathelicidin and β-defensins in eczematous lesions due to the suppressed induction effect of interleukin (IL)-4 and IL-13 on antimicrobial peptides, thereby increasing patients’ susceptibility to microbial infections (15–17).

The immune barrier plays a critical role in preventing infections, however, hyperactivation of the immune barrier may lead to an allergic reaction. Key cytokines expressed in the immune barrier include pro-inflammatory cytokines such as tumor necrosis factor-alpha (TNF-α), IL-1 and IL-6. Langerhans cells found in the barrier engulf and process foreign substances initiating the T-cell-mediated immune response. These pro-inflammatory cytokines, when over-expressed, permeate the skin barrier by disrupting filaggrin and other structural protein's production (18–21). Immune cells such as granulocytes, which are comprised of mast cells, basophils and neutrophils are also present within this barrier. These cells contain secretory granules that contain histamine, a biogenic amine that is synthesized during the decarboxylation of histidine when exposed to histidine decarboxylase (22–24). When the other barriers are compromised, degranulation occurs, releasing histamine into the extracellular space where it is metabolized (23, 24). When high levels of histamine are released, the expression of maintenance proteins such as filaggrin and keratins are downregulated, while proteases such as cathepsins and elastase are increased, which causes an increase in skin pH and promotes the expression of pro-inflammatory cytokines (13, 25–27).

In most cases, if an eczema condition is due to an allergic response, a patch test is often used to identify allergens and irritants, involves placing the suspected products and selected allergens in small 8 mm diameter chambers consisting of either aluminum or polyethylene. These chambers adhere to the skin and remain in place for 48 h before an initial reading is taken. A second reading is recorded after 96 h and in some cases a third reading after seven days is conducted, whereafter the results are interpreted using the criteria stipulated in the International Contact Dermatitis Research Group (ICDRG). These criteria include the presence or absence of erythema, infiltration, papules and vesicles and are reported as either negative, weak, strong or extremely positive reactions (28–30).

Another test that can be conducted is a prick test, which is based on the activation of IgE antibodies rather than the appearance of symptoms as stipulated by ICDRG. Suspected allergens are placed on the skin as liquid droplets at least 2 cm apart on the anterior compartment of the forearm. A different sterile needle or lancet pierces the skin through each droplet allowing the allergen to enter the dermis. Alternatively, the allergen in small quantities can be directly injected into the dermis with both methods using histamine as the positive and saline as the negative control. After 15–20 min, the test site is examined for adverse reactions, specifically the presence of wheals (hives), with a diameter of 3 mm or larger indicating a positive reaction. If no wheals are formed where the positive control was added, this may indicate that antihistamines were taken prior to the test affecting the validity of the study (28, 31).

## 4 Types of eczema

### 4.1 Atopic dermatitis

Atopic dermatitis often referred to as atopic eczema is the most common type of eczema worldwide with a prevalence of 10%–20% in children and 3%–5% in adults (32). In 2009, Odhiambo et al. published an article based on International Study of Asthma and Allergies in Childhood (ISAAC) statistics on the prevalence of atopic dermatitis worldwide. Two age groups were targeted, with data obtained from multiple centers distributed across different countries. Data obtained for ages six and seven were from 143 centers and 60 countries, while data for ages 13 and 14 were obtained from 230 centers and 96 countries. The data indicated that Africa had a resulting prevalence of 35.3% between six and seven years of age and 30.8% at ages between 13 and 14 years in 2009 with both groups frequently experiencing mild cases (9.3% and 12.8%) compared to severe cases (2.7% and 2.8%) (33).

#### 4.1.1 Etiology and immune system involvement

The exact etiology of atopic dermatitis is unknown, however two main hypotheses have been proposed, namely, the outside-in and inside-out hypothesis. The inside-out hypothesis states that the condition is due to an immunological defect, which may cause the patient to become sensitive to immunoglobulin E (IgE) (34). This hypothesis involves an imbalance in either T-helper (Th) 1 or Th-2 lymphocytes, depending on the prevalence of the condition and the age of the patient (35–37).

Patients with an acute form of the condition have been reported to have an imbalance of Th-2 and Th-22 lymphocytes, which enhances the production of IL-4, IL-5, TNF-α and IL-13 (37, 38). These cytokines promote IgE antibodies and the production of several other cytokines such as IL-17, IL-21, IL-19, IL-25 and IL-33, and affect the expression of filaggrin and ceramides responsible for maintaining the epidermal barrier (25, 34, 39). Patients with a chronic form of atopic dermatitis have been reported to have a Th-1 lymphocyte imbalance, which enhances the production of interferon-gamma (IFN-γ), IL-8, TNF-α and IL-12 (38). Of these cytokines, IL-8 and IFN-γ suppress the activation of Th-2 lymphocyte reducing the expression of IgE to normal levels and promoting other cytokines such as IFN-alpha (IFN-α) and IL-1 beta (IL-1β) (35, 36, 40).

The outside-in hypothesis proposes that atopic dermatitis is due to a skin barrier disruption caused by genetic mutations or environmental changes that lead to sensitization (39). Disruptions such as the downregulation of claudins and a skin pH of 6 affect the physical and biochemical barrier of the skin causing tight junctions to become dysfunctional interfering with lipid production, thereby reducing the expression of beta-glucocerebrosidase (β-GlcCer'ase), acidic sphingomyelinase (aSMase) and phospholipase A2 (sPLA2), while promoting the production of serine proteases (12, 13). This hypothesis involves the reduction of functional filaggrin due to a loss-of-function mutation in the filaggrin gene, which increases transepidermal water loss (TEWL) and affects the keratin-cytoskeleton causing skin dryness (12, 35, 41).

### 4.2 Contact dermatitis

Contact dermatitis is a type of eczema that develops when an individual is exposed to irritants such as toxic chemicals, metal ions and small reactive molecules that modify skin proteins resulting in an immune response. The global prevalence of contact dermatitis varies, with the condition reported as the third most common skin disease in Europe in 2018, however, the prevalence of the condition could be underestimated as contact dermatitis is often misdiagnosed as atopic dermatitis (42). Statistics relating to the prevalence of contact dermatitis across African countries is scarce as most research has focused extensively on Western ethnic groups (2).

There are various forms of contact dermatitis including irritant, allergic and protein contact dermatitis. Irritant contact dermatitis (ICD) is the most common form, is an inflammatory skin condition caused by chemical or physical injuries predominantly associated with detergents, solvents, acids and alkali through non-immune mechanisms (2, 42). Reactions that initiate ICD could either be generic (intrinsic) or due to environmental (extrinsic) factors, and the intensity of the condition can vary depending on the duration and concentration of the irritant (42). An acute form of ICD develops within minutes to hours once the skin has been exposed to a potent irritant with manifestations such as skin erythema, oedema and keratinocyte vesiculation occurring (2). Chronic forms of the condition develop gradually as the patient is exposed to mild irritants with symptoms including hyperkeratosis, lichens and cracked skin (2, 42).

Allergic contact dermatitis (ACD) occurs when the skin is exposed to a hapten or small molecules including metal substances such as nickel, fruits, perfumes and other products resulting in an immune response (2, 43). Protein contact dermatitis (PCD) occurs when proteins, often released from plants or animals, with a molecular weight of more than 10,000 daltons, initiate an immune response that is often unrelated to haptens (2, 44). Since PCD has similar clinical manifestations as other types of eczema and due to its high molecular weight, PCD is often diagnosed with a prick test instead of a patch test using the products the patients suspect may be causing the condition (44).

#### 4.2.1 Etiology and immune system involvement

All three forms of contact dermatitis involve the disruption or permeability of the skin barrier, which allows these irritants, allergens or high molecular weight proteins to enter the skin. Once entered, T-cell-mediated delayed hypersensitivity reaction occurs, allowing antigens to be produced by the body leading to sensitization. Once re-exposed to the same irritant, halogen or proteins, these antigens initiate a T-cell-mediated immune response (43).

Peptidergic and nonpeptidergic neurons have been associated with the pathophysiology of ACD due to their effect on the immune system once haptens are exposed to the skin. Peptidergic neurons are associated with initiating and maintaining inflammation by activating mast cells and promoting Th-2 priming, while nonpeptidergic neurons sustain and amplify the itching sensation (45, 46). Transient receptor potential cation channel subfamily V member 1 (TRPV1), present on peptidergic neurons, promote dendritic cell migration causing Th2 priming. This promotes an inflammatory response that activates nonpeptidergic neurons. Once active, these neurons secrete IL-31, which amplify inflammation and the itching response (46). Alink between TRPV1 and cannabinoid-mediated modulation has been established whereby the activation of cannabinoid receptor 1 (CB1) and CB2 decreases the activation of TRPV1, thereby reducing inflammation and pruritus (47). A potential targeted therapy for ACD could involve the enhanced activation of CB1 and CB2 receptors, while suppressing TPRV1.

### 4.3 Dyshidrotic eczema

Described by Tilbury Fox in 1873, dyshidrosis, commonly known as dyshidrotic eczema, is a type of eczema characterized by the formation of pruritic vesicles or small blisters located on the palms, soles and lateral sections of the fingers where a high density of sweat glands are located (2, 48, 49). These vesicles are 1–2 mm in size initiate an itchy sensation and persist for two to three weeks with reoccurring intervals leading to the eruption and subsequent vesicular development further aggravating the itching sensation (48).

Accounting for 5%–20% of eczema cases that develop on the hand, limited information regarding the potential etiology of dyshidrotic dermatitis is available. Common factors associated with the initiation of the condition include sensitivity towards various metals such as nickel and cobalt, fungal infections specifically those caused by dermatophytes, exposure to intense light (photoinduction) and intravenous immunoglobulin therapy (48, 50).

#### 4.3.1 Hypothesized etiology and immune system involvement

The etiology of dyshidrotic dermatitis has been linked to the exposure of metal ions indicating that this type of eczema may be a subtype of systemic contact dermatitis, similar to PCD. A study conducted by Stuckert and Nedorost evaluated the correlation of dyshidrotic dermatitis with the ingestion of nickel, cobalt and chromium salt. The authors found that 25% of the 202 patients experienced symptoms associated with dyshidrotic dermatitis after ingesting the metal ions, however, none of the patients displayed a positive patch test against these allergens. In addition, some patients experienced flareups without being exposed to the metal ions (51). Further investigation into the potential etiology of the condition should be conducted, specifically to determine its driven by an increase in IgE levels and to identify the potential triggers. In addition the potential mechanism of action for those that displayed flareups when exposed to the metal ions should be explored.

### 4.4 Asteatotic dermatitis

Asteatotic dermatitis, commonly known as xerosis, is a skin condition that predominantly occurs in elderly patients affecting approximately 70% of the population group over the age of 60 years (10). Common symptoms associated with this type of eczema include dry, rough and scaling skin that is itchy and inflamed (10, 52). One of the main causes of dry skin is the increase of TEWL due to a decrease in the presence of fatty acids or lipids within the stratum corneum (10).

#### 4.4.1 Hypothesized etiology and immune system involvement

The itching sensation experienced by asteatotic dermatitis patients could be due to C-fibers that transfer the sensation from the stimulated free nerve endings, located near the skin's surface, to the spinal cord and then to the cerebral cortex, whereby the sensation is processed (52). The C-fibers release substance P from the terminals, which bind to mast cells causing degranulation promoting the release of IL-1, IL-2, IL-4, TNF-α and histamine causing neurogenic inflammation (53).

This aggravated itching sensation further damages the skin allowing bacteria and allergens to penetrate the skin causing ICD and infections (10). As of current, most research on the link between substance P and eczema is conducted on atopic dermatitis and not on asteatotic dermatitis. Moreover, limited information on available treatments for asteatotic dermatitis that targets inhibition of substance P to reduce neurogenic inflammation has been found.

### 4.5 Nummular eczema

In 1854, Marie Guillaume Alphonse Devergie described nummular eczema as small round patches located on the surface of the limbs and trunk with a thin circumference that is often itchy and takes several months to recover (54). This rare type of eczema is often present in patients between the ages of 18–64 with its etiology associated with environmental factors, allergens and nutritional factors, however, the exact cause of the condition remains underexplored (55, 56).

#### 4.5.1 Hypothesized etiology and immune system involvement

There is speculation that the etiology of the condition is due to the overexpression of IL-1, IL-2 and IL-6. A study conducted by Vyu et al. evaluated the effect of the condition on the production of IL-10, IL-12 and IL-17 by conducting immunoassays on 38 patients who had nummular eczema. During this study, the authors reported that IL-12 and IL-17 were over-expressed in nummular eczema patients compared to the control, while IL-10 was downregulated indicating a potential correspondence with these cytokines and IL-1, IL-2 and IL-6 (55).

Previously IL-17 has been associated with the reduced expression of filaggrin and ceramides responsible for maintaining the epidermal maintenance barrier, while the expression of IL-12 induces the expression of Th-1 cells which promotes the release of other cytokines including IL-8 (25, 34, 38, 39, 57). In a review published by Liu et al., and Xu and Cao, the overexpression of IL-12 and IL-17 promotes the expression of IL-6, while IL-1 may induce innate IL-17 production (58, 59). The downregulation of IL-10 may promote the expression of IL-2 as Couper et al. reported that IL-10 reduced the expression of IL-2 when a patient was exposed to an infection (60).

### 4.6 Seborrheic dermatitis

Seborrheic dermatitis is a type of eczema located near high density of sebaceous glands located on the scalp, face, chest, back and groins. Diagnosis of the condition is mostly based on the location and the presence of lesions, which are often greasy (49, 61). The prevalence of seborrheic dermatitis varies between age groups and countries with most demonstrating a high occurrence in infants, adolescents and young adults, with a notable rise in cases among patients older than 50 (Table 1) (62, 63). Polaskey et al. conducted a literature search on the prevalence of seborrheic dermatitis and found that studies from the year 1788–2024 reported the prevalence of the condition in nine countries. South Africa displayed the highest prevalence at 8.82%, whereas African countries such as Nigeria and Egypt displayed a prevalence of 3.61 and 3.52%, respectively (62).

#### 4.6.1 Hypothesized etiology and immune system involvement

The etiology of seborrheic dermatitis is unknown but several factors have been shown to contribute to the condition's development including hormone levels, cytokines that have an immunological effect including increasing the immunoglobulin G (IgG) serum levels and comorbid conditions such as Parkinson's disease, human immunodeficiency virus (HIV) and acquired immunodeficiency syndrome (AIDS). Studies have also indicated that *Malassezia* fungal species have a high association with the condition. *Malassezia* species specifically *Malassezia furfur* and *M. ovalis* are lipid-dependent opportunistic fungi that often form part of the skin's commensal microbiome and are predominantly found in seborrheic regions such as the scalp and face (63–66).

During the first week of life, *Malassezia* species rapidly colonize the skin, forming between 13% and 50% of the skin's commensal microbiome. During puberty, there is an increase in sebaceous lipids which promotes the colonization of *Malassezia.* Studies suggest that the high colonization of *Malassezia* species triggers an immunological response promoting symptoms associated with seborrheic dermatitis (67–69). A study conducted by Suzuki et al. indicated that when *Malassezia* species are exposed to monocytic cell lines (THP-1) and granulocytic cell lines (HL-60), pro-inflammatory cytokines such as IL-8 and IL-1α are stimulated (70). Produced by keratinocytes to initiate inflammation, IL-1α has been found to activate nuclear factor kappa beta (NF-κB) as well as promote the expression of IL-8 (71, 72). On the other hand, IL-8 promotes cell migration and the recruitment of monocytic cells that differentiate into macrophages synthesizing other pro-inflammatory cytokines including TNF-α (73).

Table 1 contains a summary of each type of eczema with regards to their prevalence, symptoms and their potential etiology and pathogenesis.

## 5 Socioeconomic burden of eczema

A literature review by Drucker et al. examined qualitive studies assessing the socioeconomic impact of atopic dermatitis in America using data provided by the National Eczema Association and additional sources. The authors noted that the quality of life of atopic dermatitis patients were greatly affected due to disease-related symptoms. These symptoms, specifically pruritus, affected everyday activities such as clothing selection (35%) and social engagement (25.5%) (74).

Another study reported that patients with atopic dermatitis are conflicted when choosing their careers due to symptoms associated with the condition. The authors noted that during a 2002 survey, 38% of the participants reported a decline in educational and occupational opportunities due to their condition. Moreover, many atopic dermatitis patients tend to avoid occupations such as food service, cleaning, maintenance, healthcare and other opportunities that are associated with a high risk of developing dyshidrotic dermatitis, thereby limiting their career options (75).

Although the factors described above are associated with the direct social burden of eczema, many studies focus on the direct and indirect economic impact of eczema. According to a review by Abramovits et al., the authors mentioned the cost of atopic dermatitis in several countries such as Australia, the United Kingdom and the United States between 1995 and 1997 (76). The authors indicated that the cost per year for Australia, the United Kingdom and the United States was $A1,142–6,099, £250 per child and USD 609 per patient, respectively. Due to inflation, these costs are equivalent to approximately $A2,507.23–13,390.21, £511.93 per child and USD 1,279.92 per patient in 2025 (76). Limited information on the socioeconomic impact on South Africa can be found, however, based on the equivalent costs of Australia, the United Kingdom and the United States it is estimated that the condition could cost between ZAR12,766.33–ZAR23,852.40 per patient and ZAR29,990.79–ZAR160,169.98 per year. These out-of-pocket costs are dependent on the patient's healthcare plan as all South Africans are entitled to public healthcare services, however, many choose to pay for private healthcare. This is due to various advantages associated with private healthcare services as these services are often supported by external funders, providing hospitals and doctors with advanced training and medical equipment. These services are often partly or fully covered by those who have private medical aid but are out-of-pocket for those that utilize public services (77).

Moreover, with regards to eczema-based therapies, most of these private healthcare services partly or fully cover doctoral consultations, referred to specialists and prescribed FDA approved conventional treatments, dependent on the patient's healthcare plan. Conventional treatments that are FDA approved for eczema, regardless of the type, are referred to as in-labelled treatments, while those that are FDA approved for other conditions but may be used for eczema, are referred to as off-labelled products, however, depending on the patient's service plan both in- and off-labelled treatments may be out-of-pocket. Any out-of-pocket costs incurred by the patient are not normally reimbursable.

Many eczema related expenses are covered by both private and public healthcare there are additional expenses that are not included such as moisturizers and emollients, certain types of medication, phototherapy, inpatient treatments and travel expenses. A study conducted by Zink et al. evaluated the additional out-of-pocket expenses of 1 189 European participants. The authors reported that the mean extra expenses per month in 2017 was €77.26 with most spending a mean cost of €27.63 on emollients and moisturizers, €17.74 on medication that is not included in their health plan and €8.48 on alternative treatments such as phototherapy. The authors further reported that patients spent a mean of 18% extra on personal hygiene, 8% on clothing and food and 7% bedding and gloves resulting in an overall yearly cost of €927.12 (78).

Reports on the socioeconomic impact of other types of eczema are not widely available. Gladys et al. reviewed the cost of care for dyshidrotic dermatitis patients utilizing data from the IBM MarketScan® Commercial Database situated in the United States. The study reported that 34,932 people in the United States were diagnosed with dyshidrotic dermatitis in 2018. The authors also reported on the mean cost per age group based on physician visits and treatments as well as the cost of each type of medication and procedure currently available (79).

During the study, the authors reported that the highest costs were associated with age groups between 0 and 17 (USD 110.87) and 35–44 years of age (USD 111.88). Furthermore, the authors found that the most expensive treatments used by patients were ultraviolet light A (USD 214.39), intramuscular corticosteroids (USD 132.33), azathioprine (USD 114.75), oral corticosteroids (USD 114.47) and medium potency topical corticosteroids (USD 111.75) (79). Due to inflation, the equivalent mean costs as well as the estimated cost in South Africa based on the 22nd of April 2025 exchange rate have been provided (Table 2).

**Table 2:** Equivalent financial cost of treatments, procedures and overall mean cost per age group of patients suffering from dyshidrotic dermatitis in the United States in 2025 based on the information provided by Gladys et al. (79) as well as the estimated South African cost based on the 22nd of April 2025 exchange rate (51).

Types of treatments used on different age groups	Range	Equivalent cost due to inflation (USD)	Estimated cost in South Africa (ZAR)
Age groups	0–17	141.57	2,637.30
	18–34	137.74	2,565.95
	35–44	142.84	2,660.95
	45–54	139.02	2,589.79
	55–64	136.47	2,542.29
Topical corticosteroids	Super-high potency (Group 1)	136.47	2,542.29
	High potency (Group 2)	139.02	2,589.79
	High potency (Group 3)	132.64	2,470.94
	Medium potency (Group 4)	142.84	2,660.95
	Lower-mid potency (Group 5)	137.74	2,565.95
	Low potency (Group 6)	133.92	2,494.78
	Least potent (Group 7)	137.74	2,565.95
Oral corticosteroids		145.39	2,708.46
Intramuscular corticosteroids		168.35	3,061.66
Azathioprine		146.67	2,732.30
Mycophenolate		140.29	2,613.45
Procedures	Ultraviolet A	272.93	5,084.39
	Ultraviolet B	116.06	2,162.07

## 6 Conventional treatments

### 6.1 Corticosteroids

Corticosteroids, also known as glucocorticosteroids, are common in-labelled treatments used to alleviate inflammation and immune-based conditions and have been prescribed to patients since the 1950s (Table 3). Corticosteroid based applications currently available include ointments, gels and creams, tablets and pumps, which readily diffuse into the cell membrane binding to glucocorticoid receptors (80, 81). Once bound, the corticosteroids are translocated into the nucleus where the compound binds to the glucocorticoid response element and regulates the expression of pro-inflammatory cytokines such as IL-10, IL-4, IL-13 and inhibitor of nuclear factor kappa beta (IκB) and anti-inflammatory cytokines, including IL-2, TNF-α, IL-1β and cyclooxygenase-2 (COX-2) (80, 82). Corticosteroids potency levels are classified based on various factors depending on the classification system used in each country. Table 4 depicts some of the main corticosteroids and their potency levels as described by Stacey and McEleney (83). This classification is used to regulate what type of treatment is given to the patient based on their condition, the duration of the treatment and the age group (83). There are three classification systems namely the United States seven-category system, the United Kingdom's four-category classification and the anatomical therapeutic classification (ATC) (84).

**Table 3:** Recommended in- and off-labelled conventional treatment for various eczema types based on evidence-based studies and clinical trials.

Type of treatment	Mode of action	Evidence-based studies and clinical trials	Recommended eczema types	Approval (in-label or off-label)	References
Corticosteroids					
All treatments classified as corticosteroids	Regulates the expression of various cytokines such as interleukin (IL)-10, IL-4, IL-13, IL-2, tumor necrosis factor alpha (TNF-α), IL-1 beta (IL-1β), cyclooxygenase-2 (COX-2) and inhibitor of nuclear factor kappa beta (IκB).	Few studies on the effect of corticosteroids have been reported, however, these treatments are often used by physicians.	Often used as the gold standard and prescribed to patients with any type of eczema subtype.	Approved by the Food and Drug Administration (FDA) and by European countries for the use of all types of eczema (in-label).	(80, 82, 174)
Immunosuppressive agents					
Azathioprine	Reduce the number of epidermal Langerhans cells, blood leukocytes and the synthesis of immunoglobulin M (IgM) and IgG.	Evidence-based studies • Significantly lowered the synthesis of both immunoglobulins by 33.4 and 40.9%, respectively after four months of use. • Clinical trials • Significantly reduced Scoring of Atopic Dermatitis (SCORAD) by 26%–39% after 12 weeks.	Contact dermatitis, atopic dermatitis and potentially seborrheic dermatitis.	Not approved in European countries or in America for all types of eczema (off-label).	(85, 86, 148) (150, 174)
Mycophenolate	Potentially inhibit the proliferation of T- and B-lymphocytes.	Evidence based: • After 12 weeks IFN-γ significantly increased from 2.40 to 3.00 pg/mL (*p* < 0.02), while IL-10 decreased by 65.5% (*p* < 0.001). • Clinical trials: • After four weeks, symptoms associated with dyshidrotic dermatitis significantly reduced. • Post-SCORAD scores were reduced by a significance of *p* < 0.0002 when used on atopic dermatitis patients for a period of approximately seven weeks.	Used on dyshidrotic patients, atopic dermatitis and potentially for contact dermatitis.	Not approved in European countries or in America for all types of eczema (off-label).	(88–90, 153, 174)
Ciclosporin A	Reduces the transcription of IL-2 preventing the activation of T cells.	Clinical trials • Significantly reduced atopic dermatitis by 58% after an initial treatment period of 6 weeks. • Reduced dyshidrotic dermatitis by 54% after an initial treatment period of 6 weeks.	Atopic dermatitis and dyshidrotic dermatitis	Approved in most European countries for the treatment of severe atopic dermatitis (in-label) but not approved in America for any type of eczema (off-label).	(91, 92, 174, 175)
Phototherapy					
Ultraviolet (UV) A-1	Inhibits the expression of T helper (Th)-2 cytokines while upregulating IL-10.	Clinical trials: • UVB narrowband reduced the SCORAD of atopic dermatitis patients by 50.8%. • PUVA reduced the SCORAD of atopic dermatitis patients by 65.7%.	Atopic dermatitis	All types of phototherapies are approved in America to treat atopic dermatitis only (in-label), while these treatments are off-labelled in European countries.	(95–98, 145, 176–179)
UVB narrowband	Promote the production of pro-inflammatory cytokines including prostaglandins, TNF-α, IL-10 and alpha-melanocyte stimulating hormone (α-MSH). Downregulates the expression of intercellular adhesion molecules-1 (ICAM-1).				
UVB broadband					
UVA-1 with psoralens (PUVA)	Kill T cells that are associated with the itching sensation.				

**Table 4:** Potency levels of main corticosteroid actives based on a study conducted by Stacey and McEleney (83).

Potency levels	Active ingredient	Molecular structure
Super high potency (Group 1)	Fluocinonide 0.1%	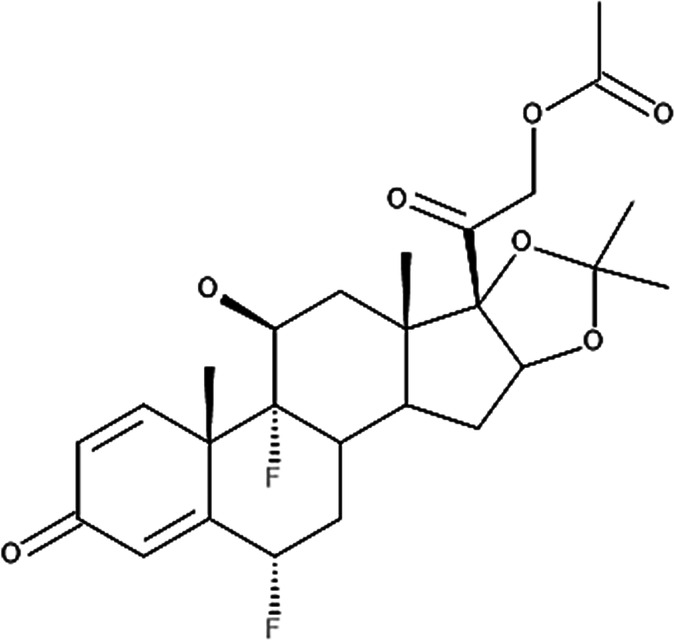
	Clobetasol propionate 0.05%	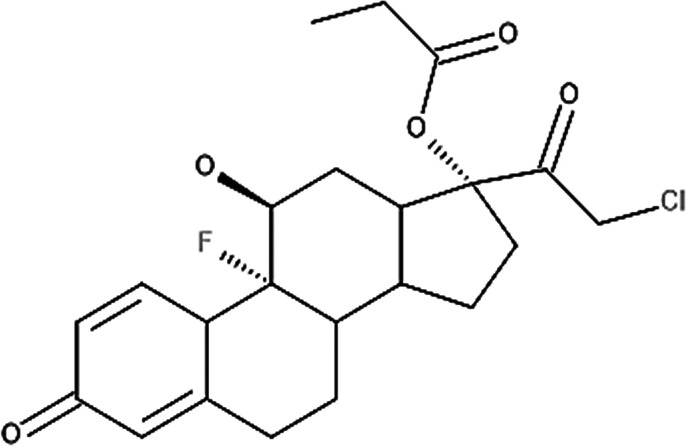
High potency (Group 2)	Amcinonide 0.1%	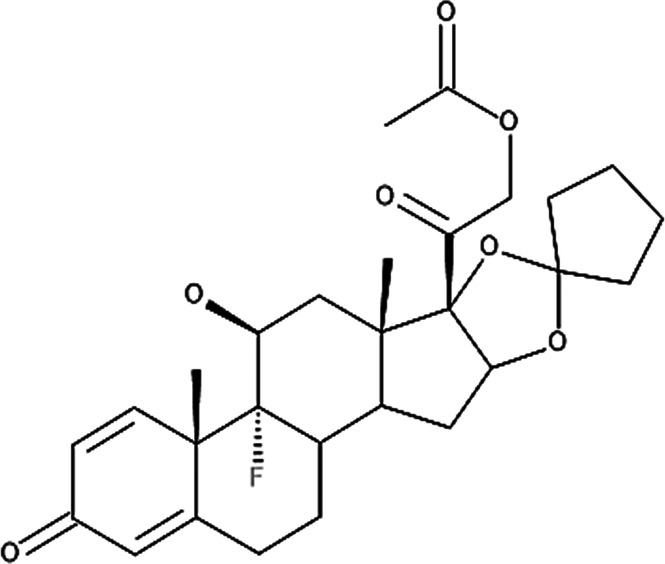
	Desoximetasone 0.25%	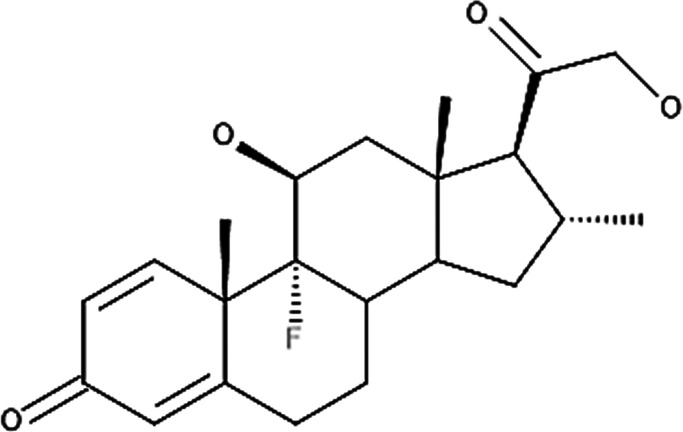
High-medium potency (Group 3, 4 and 5)	Betamethasone dipropionate 0.05%	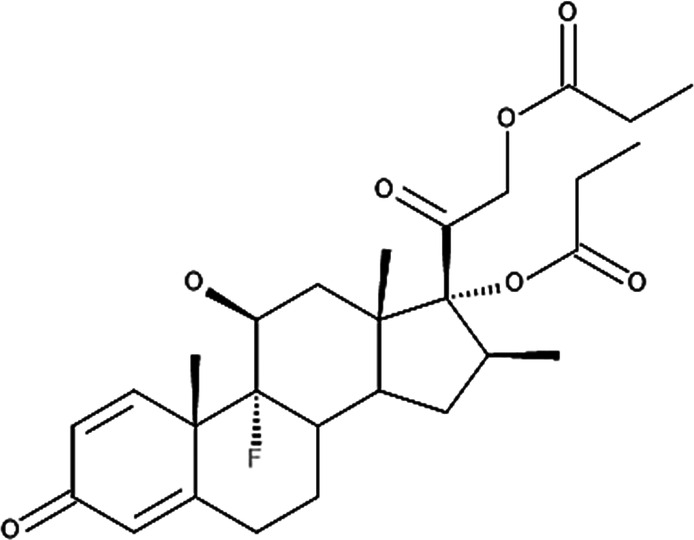
	Fluocinolone acetonide 0.025%	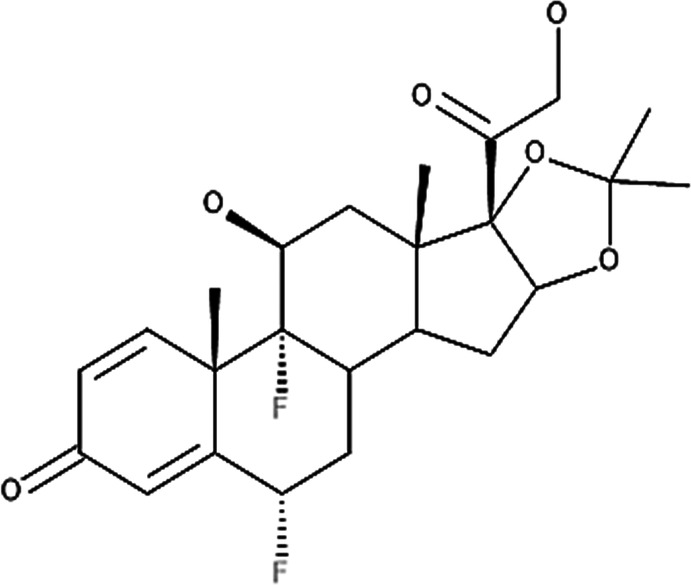
	Mometasone furoate 0.1%	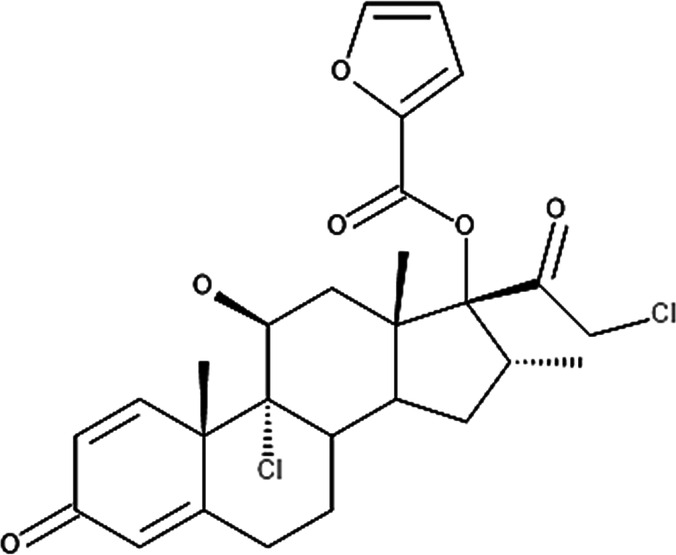
Low potency (Group 6 and 7)	Desonide 0.05%	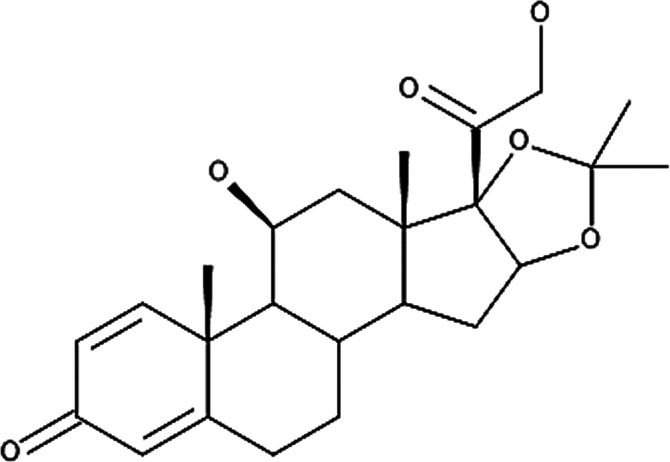
	Hydrocortisone 1%	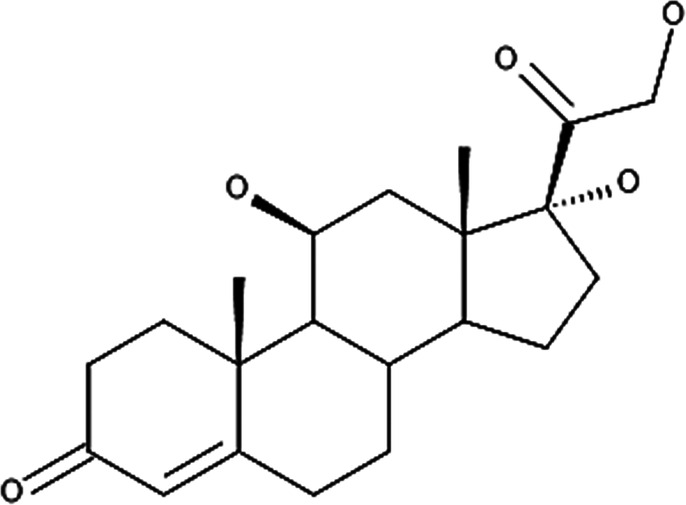

The United States seven category system focuses on the active ingredient's vasoconstrictive effect, indicating high cutaneous absorption and clinical effectiveness by evaluating whether the mean Scoring of Atopic Dermatitis (SCORAD) score is significantly lower than the control. Once these have been taken into account, the ingredients are ranked from one being the most potent, indicating a significant effect against eczema with high vasoconstrictive potential, and seven being the least (84). The United Kingdom's four-category classification ranks the treatment's potency based on their vasoconstrictive properties and effective concentration that significantly reduces the SCORAD score, whereby four is the most potent due to high constrictive potential and low concentration and one is mild. The last classification uses a similar potency rank as the United Kingdom, however, ATC classifies dermatologic corticosteroids and not those taken orally and focuses on clinical studies conducted on the corticosteroid and not on the final formulation (84).

### 6.2 Immunosuppressive agents

Azathioprine, a derivative of 6-mercaptopurine, is an off-label immunosuppressive agent that was first made available in the 1960s (Table 3) (85). The mechanism of action is not completely understood, however, it has been shown to reduce the number of epidermal Langerhans cells and blood leukocytes as well as reduce the synthesis of IgM and IgG (85, 86). The main concern associated with the use of azathioprine is that high and prolonged use may be mutagenic and carcinogenic, therefore increasing the risk of skin cancers including squamous cell carcinoma, malignant neoplasms and Kaposi sarcoma. Other skin conditions associated with the extended use of azathioprine include scabies and dermatomycoses (85).

Another off-label immunosuppressive agent that is used is mycophenolate. In 1913, Alsberg and Black isolated mycophenolic acid (MPA) as a fermentation product from *Penicillium stoloniferum*. This lipid-soluble acid was found to have antibacterial, antifungal and antiviral activity, however, the use of this acid was unfavorable due to its carcinogenic potential. In 1995, a semisynthetic morpholinoethyl ester of mycophenolic acid, also known as mycophenolate mofetil (MMF), was developed, which was approved by the U.S. Food and Drug Administration (FDA) as an immunosuppressive agent due to its higher bioavailability and lower side effects than MPA (87).

When ingested, MMF is converted to MPA, which inhibits inosine monophosphate dehydrogenase preventing the conversion of inosine monophosphate to xanthosine monophosphate reducing the availability of guanine nucleotides. This reduction in guanine nucleotides reduces purine nucleotide synthesis, which invertedly inhibits the proliferation of T- and B-lymphocytes as these lymphocytes are dependent on this synthesis. The ethyl ester attached to MMF ensures that the half-life of MPA is reduced significantly, lowering its carcinogenic potential (88–90).

Ciclosporin A, an off-label cyclic oligopeptide immunosuppressor, suppresses the cytoplasmic calcineurin phosphatase activity by reducing the transcription of IL-2, preventing the activation of T cells (91, 92). This immunosuppressor is often used as a first line treatment for patients with severe atopic dermatitis, however, as of 2021 this immunosuppressor has only been approved in Europe and Japan with limitations due to risk of hypertension and nephrotoxicity when used over an extended period (93).

### 6.3 Phototherapy

Phototherapy is considered a second line of treatment that is often recommended to adults who have atopic dermatitis (Table 3). First reported to treat psoriasis in 1925, this type of treatment only became popular in 1978 after the General Hospital in Massachusetts published trials on the effectiveness of phototherapy in eczema patients. Since then, various forms of phototherapy have emerged including ultraviolet light (UV) A and UVB. Ultraviolet light A is subdivided into UVA-1 (340–400 nm) and UVA-2 (320–340 nm) of which UVA-1 is more frequently used, while UVB is subdivided into broadband UVB (BBUVB) with a wavelength range of 290–320 nm and narrowband UVB (NBUVB) with a range of 311–313 nm (94, 95).

When the skin is exposed to UV radiation, various structures within the skin will either absorb or reflect the rays (96). With a wavelength of 320–400 nm, UVA is absorbed in the dermal layer of the skin affecting various skin structures, cells and immune cells including dermal fibroblasts, dendritic cells, endothelial cells, T lymphocytes, mast cells and granulocytes (97). Ultraviolet light B has a shorter wavelength range of 280–320 nm and penetrates the epidermal layer affecting keratinocytes, melanin and Langerhans' cells (96).

Molecules that can absorb UVA and UVB are classified as chromophores and are altered when exposed to these rays. Once modified these photoproducts initiate various responses within the body including inflammation, immunosuppression and apoptosis. One of the main chromophores is DNA, of which UVB is more likely to be absorbed, while UVA initiates the production of reactive oxygen species causing the DNA to indirectly degrade (95). Unlike other UV rays, DNA does not absorb UVA-1, instead the rays are absorbed by immune cells, thereby inducing apoptosis and inhibiting the expression of Th-2 cytokines including IL-5, IL-13 and IL-31 as well as upregulating IL-10 (95, 97).

A combination of UVA-1 with psoralens (PUVA), which are photosensitizing substances that are either taken orally, can be applied topically or through balneotherapy (98). After administering psoralens, the patient is exposed to UVA. The psoralens act as a type of chromophore enabling UVA to bind to the DNA, initiating apoptosis of T cells that have migrated into the skin (95). The mechanism of action associated with PUVA involves DNA damage, depletion of nucleotides present in blood leukocytes as well as T-cell death which has been associated with the itching sensation (98).

UVB can be recommended for children and pregnant women due to the limited penetration potential of the rays and because no additional photosensitization is required (95). Chromophores that absorb UVB include proteins such as keratin, melanin, collagen, DNA and elastin. These photoproducts, once exposed to UVB, initiate an immune response, whereby the production of pro-inflammatory cytokines, including prostaglandins and TNF-α, and anti-inflammatory factors, such as IL-10, alpha-melanocyte stimulating hormone (α-MSH) and prostaglandin E2 (PGE2) is enhanced (96). UVB has been shown to downregulate the expression of intercellular adhesion molecules-1 (ICAM-1). This molecule has been linked to both psoriasis and atopic dermatitis as ICAM-1 is often over-expressed in patients suffering from these conditions (97).

Phototherapy is used as an alternative to corticosteroids, however, various adverse effects have been reported, specifically the high risk of developing cancer when utilizing the treatment over a long period of time. These adverse effects include erythema, burning, pigmentation disorders, gastrointestinal intolerance to psoralens and pruritus. Skin ageing can occur when patients are exposed to UVA-1 as this ray penetrates the dermal layer affecting elastin and collagen production. Other adverse effects include nonmelanoma and melanoma skin cancer, headaches, nausea and cataract formation when applying psoralens on the face (94, 95).

### 6.4 Targeted therapy

In 2017, the biologic dupilumab was approved for the treatment of patients with atopic dermatitis, whereas small molecules were approved in February 2025 (99). These in and off-labelled targeted therapies have been used to manage moderate to severe atopic dermatitis cases for patients that have had inadequate response to the above-mentioned conventional treatments. As present, most available biologics and small molecule therapies that are available have been evaluated in atopic dermatitis patients, with only a few studies conducted on individuals with dyshidrotic eczema. The following section provides an overview of targeted therapies currently used.

#### 6.4.1 Biologics

Biologics are synthetic compounds that are extracted or sourced from various biological materials including cell lines, microbial agents, plants and fungi, and are categorized as monoclonal antibodies, receptor modulators and modulators of enzymes (100). The most well-known monoclonal antibody is dupilumab, as it was the first biologic agent approved for the treatment of atopic dermatitis, however, there are similar biologics to dupilumab that utilize other mechanisms of action including tralokinumab and lebrikizumab, which have been approved for moderate to severe atopic dermatitis (99). Dupilumab is a human IgG4 monoclonal antibody, administered through subcutaneous injection, developed by Regeneron Pharmaceuticals and Sanofi, that is currently approved by the Food and Drug Administration (FDA) for use as a treatment of moderate to severe atopic dermatitis (101, 102).

This monoclonal antibody inhibits IL-4 and IL-13 signal transductions as it competitively binds to the cytokine's respective receptors. Dupilumab also targets downregulates inflammatory mediators and epidermal proliferation, while upregulating structural and epidermal barrier proteins (103). Tralokinumab and lebrikizumab are also IgG4 monoclonal antibodies, however tralokinumab binds to IL-13 preventing this cytokine from binding to the IL-13 receptor, while lebrikizumab blocks IL-13 signal by binding to soluble IL-13 at an epitope preventing the formation of IL-4 receptor alpha (IL-4Rα)/IL-13 receptor alpha 1 (IL-13Rα1) heterodimeric receptor complex (99). Another monoclonal antibody that was approved in February 2025 by the European Medicines Agency (EMA) is nemolizumab, a human IgG2 monoclonal antibody, also administered through subcutaneous injection, that inhibits IL-31 signal transduction by binding to the IL-31 receptor, thereby reducing pruritus, inflammation and epidermal dysregulation (Table 5) (99).

**Table 5:** Recommended in- and off-labelled targeted treatments for eczema subtypes based on their mode of action.

Type of treatment	Mode of action	Recommended eczema types	Approval (in-label or off-label)	References
Biologics				
Dupilumab	• Inhibits interleukin (IL)-4 and IL-13 signal transductions as it competitively binds to the cytokine's receptors. • Downregulates inflammatory mediators and epidermal proliferation, while upregulating structural and epidermal barrier proteins.	Atopic dermatitis (chronic and acute)	Approved in America through the Food and Drug Administration (FDA) as a treatment for atopic dermatitis for patients above the age of 6 months (in-label).	(101–103, 174, 177)
Tralokinumab	Prevents IL-13 from binding to the IL-13 receptor.	Atopic dermatitis	FDA approved to treat atopic dermatitis patients above 12 years of age (in-label).	(99, 174, 177)
Lebrikizumab	Blocks IL-13 signal by binding to the IL-4 receptor alpha (IL-4Rα)/IL-13 receptor alpha 1 (IL-13Rα1) heterodimeric receptor complex.	Atopic dermatitis	Approved in Europe to treat patients 12 years and older that have atopic dermatitis (in-label).	(99, 174)
Nemolizumab	Inhibits IL-31 signal transduction by binding to the IL-31 receptor.	Atopic dermatitis	Approved in Japan to treat pruritus, while in 2025 Europe approved nemolizumab use on atopic dermatitis patients (in-label).	(99, 174)
Small molecules				
Delgocitinib	Targets the entire Janus kinase (JAK) family that consists of JAK1, JAK2, JAK3 and tyrosine kinase 2 (TYK2).	Atopic and dyshidrotic dermatitis	Approved in Japan to treat atopic dermatitis (mostly off-label).	(107, 109, 174)
Ruxolitinib	Inhibits JAK1 and JAK2	Atopic dermatitis	FDA approved for patients above the age of 12 (in-label).	(108, 110, 177)
Gusacitinib	Targets the production of various cytokines including interleukin 1 beta (IL-1β), IL-10, IL-17, IL-4, IL-13 and IL-33	Atopic and dyshidrotic dermatitis	Used off label in both America and Europe.	(107, 111, 112)
Upadacitinib	Affects the production of T helper (Th)-2 related cytokines, including IL-4, IL-5 and IL-31.	Atopic dermatitis	Approved in both America and Europe to treat atopic dermatitis (in-label).	(113, 114, 174, 177)
Abrocitinib	Affects the production of Th-2 and targets IL-4Rα.	Atopic dermatitis	Approved in Europe, Japan and America for the treatment of moderate to severe atopic dermatitis in patients older than 12 years old (in-label).	(115, 116, 174, 177)
Baricitinib	Inhibits cytokine signaling associated with Th-2 (IL-4, IL-13, IL-31), Th-17 (IL-22) and Th-1 (IFN-γ, IL-4, IL-13 and IL-31).	Atopic dermatitis	Initially approved for rheumatoid arthritis in America and thus is used as an off-label treatment. Approved in Europe for atopic dermatitis in patients above the age of 2 years (in-label).	(107, 117, 174, 177)

Biologics that are currently undergoing clinical trials that target IL-4Rα and tumor necrosis factor receptor superfamily member 4 (OX40) include rademikibart and rocatinlimab (99). Similar to dupilumab, rademikibart inhibits IL-4 and IL-13 through competitive binding. A study conducted by Zhang et al. showed that rademikibart did not cross-react with other mammalian receptors and displayed a significant (*p* < 0.01) inhibitory effect against IL-4 mediated signal transducer and activator of transcription 6 (STAT6) signaling (7.00 ± 2.50 ng/mL) in cytokine-induced splenocytes compared comparison to dupilumab (9.90 ± 2.70 ng/mL) (104). In contrast, rocatinlimab targets OX40, present on T cells. This receptor functions as a T cell costimulatory molecule that is only present on T cells that have been stimulated by the presence of an antigen and assists with differentiation as well as the induction of memory cell formation, enhancing the pro-inflammatory response (99, 105, 106).

#### 6.4.2 Small molecules

Small molecules are immune-modulating agents that target enzymes in the Janus kinase family (JAK) that are closely linked to STAT pathways. This is because JAK-STAT modulate the signaling of Th-2 cytokines including IL-4, IL-5, IL-13 and IL-31, which contribute to inflammation and pruritus and disrupt epidermal maintenance barrier proteins such as filaggrin, loricrin and involucrin (107, 108). Several JAK inhibitors have been developed including delgocitinib, ruxolitinib for topical application, gusacitinib, upadacitinib and baricitinib for oral administration (Table 5) and tofacitinib for both applications (107).

Delgocitinib is classed as a pan-JAK inhibitor as it targets the entire JAK family that consists of JAK1, JAK2, JAK3 and tyrosine kinase 2 (TYK2), and has been associated with suppressing inflammation and enhancing the skin barrier. Delgocitinib is approved in Japan for the management of atopic dermatitis, furthermore, clinical trials have shown improvement in reducing symptoms associated with hand eczema or dyshidrotic dermatitis (107, 109). Unlike delgocitinib, ruxolitinib is an FDA approved JAK1 and 2 inhibitor that is used to treat mild to moderate atopic dermatitis in patients from the age of 12 years and older and has been shown to have anti-inflammatory and anti-proliferative potential (108, 110).

Gusacitinib is an oral dual inhibitor that acts as a pan-JAK inhibitor further reducing the production of JAK spleen tyrosinase kinase (JAK-SYK). This JAK-inhibitor targets the production of various cytokines including IL-1β, IL-10, IL-17, IL-4, IL-13 and IL-33 (111). Clinical trials conducted using gusacitinib have shown to improve moderate to severe atopic dermatitis after 28 days at a dosage of 40 and 80 mg (107). Against dyshidrotic dermatitis, 80 mg gusacitinib had a 69.5% decrease in hand modified total lesion-symptom score (mTLSS) compared to the placebo (33.5%) (112). Upadacitinib is a JAK1 inhibitor that affects the production of Th-2 related cytokines, including IL-4, IL-5 and IL-31 (113). Clinical studies showed that patients aged 12–75 years showed improvement after 16 weeks when taking either 15 mg (68%) or 30 mg (74%) compared to the placebo (decreased improvement by 12%) (114).

Similar to upadacitinib, abrocitinib is an oral JAK1 inhibitor, approved in Europe and Japan in 2021 and FDA-approved in 2022 for the treatment of moderate to severe atopic dermatitis in patients older than 12 years old (115, 116). Baricitinib is a JAK1 and JAK2 inhibitor that has an additional affinity for JAK3 and TYK2 and alleviates moderate to severe atopic dermatitis by inhibiting cytokine signaling associated with Th-2 (IL-4, IL-13, IL-31), Th-17 (IL-22) and Th-1 (IFN-γ, IL-4, IL-13 and IL-31) (107, 117). Although baricitinib is used on atopic dermatitis patients, this treatment was initially approved for rheumatoid arthritis. Lastly, tofacitinib, a JAK1 and JAK3 inhibitor, is the only small molecule that can be administered orally and topically. This inhibitor suppresses similar T helper cells as baricitinib reducing the production of IL-4, IL-13, IL-22, IL-31 and IFN-γ (107).

## 7 Complementary and alternative medicine currently available

Due to the cost of conventional treatments, the adverse effects and the limited suitability for children and pregnant women, many patients favor complementary and alternative medicine (CAM), such as phytotherapy, herbs and acupuncture, however, most of these treatments are used to treat an array of conditions that have similar symptoms and are not specifically developed for eczema patients and therefore, none of these treatments abide by eczema associated guidelines (118). Moreover, the treatments mentioned fall under the alternative or complementary category, which is often self-regulated depending on the country where the product is sold and the regulatory system that is followed. These treatment's effects are often based on *in vitro* and animal orientated *in vivo* studies that have been conducted. Regardless of healthcare services, alternative treatments are more affordable than conventional or targeted due to import and research costs associated with these treatments. Furthermore, conventional treatments require prior doctoral consultations while alternatives do not, reducing their out-of-pocket costs and are easily accessible to those in rural communities.

Phytotherapy or herbalism is classified as treatments that utilize plant-derived extracts as the active ingredients to treat conditions or to use as a health-promoting agent. These plant-based extracts can either be prepared based on traditional knowledge or on research conducted on the plant (119, 120). Treatments can consist of a single extract or a mixture of extracts that work in synergy. Upon commercialization, plant extracts are standardized using a reference marker compound to verify that the composition and concentration of the extract are consistent (120). There are various phytotherapies used by eczema patients as discussed below.

### 7.1 Traditional Chinese medicine

Traditional Chinese medicine (TCM) is a form of CAM that utilizes herbs, acupuncture, massages and exercise. Most of TCM's principles are philosophically based, which is reliant on the state of the body and mind at the time of treatment, however, various treatments involving the use of herbs and acupuncture are used today to treat various conditions including eczema (121). A survey conducted in 2005 found that 30% of patients at the pediatric dermatology clinic had previously been given TCM in the past 12 months indicating that practitioners utilize this form of treatment (122).

There are multiple studies which focus on the use of a combination of Chinese herbs to assist eczema patients. One combination that has been extensively studied is PentaHerbs capsules containing *Lonicera japonica* Thunb., *Mentha canadensis* L., *Paeonia x suffruticosa* Andrews, *Atractylodes lancea* DC. and *Phellodendron amurense* Rupr (123). *In vivo* studies, both on mice and eczema patients have been conducted to evaluate the effect of PentaHerbs against eczema, allergic reactions and various cytokines (123, 124). PentaHerbs significantly reduced the number of eosinophils, neutrophils and lymphocytes present in mice (*p* < 0.05) and reduced the production of IL-4, IL-31 and IL-5 after 8 days (124). On the contrary, after administering to eczema patients, the authors found that after 12 weeks, there was no significant difference (49.7 score) in the mean SCORAD score compared to the control group (46.9 score). The authors observed that the use of corticosteroids by those administered with PentaHerbs decreased by one-third (*p* < 0.024) indicating that the treatment could be used to reduce the dependency on corticosteroids (125).

### 7.2 Kamillosan-Creme

Kamillosan-Crème is a topical treatment that contains the flower of *Matricaria recutita* L. (German chamomile) as the active ingredient. This aromatic herbaceous plant is native to Southern and Eastern Europe and Asia and has spread to a few countries including North America and North Africa. Traditionally the flowers, roots and oils of German chamomile have been used extensively to treat various ailments including inflammatory skin conditions when applied topically, gastrointestinal disorders when ingested as a tea and for colds when the aromas are inhaled (126). Due to the plant's vast properties, German chamomile is also consumed as food, used in cosmetics and disinfectants (127).

Various compounds have been isolated from the flowers of the German chamomile with the main constituents consisting of α-bisabolol, apigenin, luteolin and derivatives thereof, such as quercetin 3-glucoside and menthol (126, 128, 129). For standardization of Kamillosan-Crème and other chamomile-based products, 0.25% of apigenin 7-glucoside needs to be present in the prepared extract to be suitable for use according to the European Pharmacopoeia's recommendations as apigenin is able to inhibit the synthesis of COX-2, which promotes inflammation when expressed and its reducing effect on the production of histamine assisting with the itching sensation (Table 6) (126, 128–130). Based on the established and hypothesized mechanisms as mentioned in Section 4 and the potential biological activity of actives derived from *M. recutita* (Table 6), Kamillosan-Crème could potentially be used to treat most of the eczema subtypes, however, clinical efficacy in humans against each subtype should be considered to confirm the effectiveness of the treatment compared to conventional treatments.

**Table 6:** Secondary plant metabolites used in alternative treatments that have been shown to reduce targets associated with eczema.

Compound name	Plant species	Structure	Potential targets	Related evidence-based results	References
α-Bisabolol	*Matricaria recutita* L.	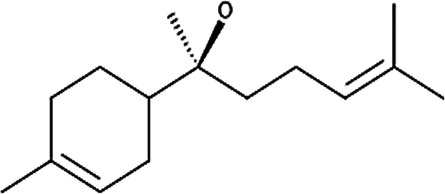	Inflammation and wound healing	• When combined with heparin, inflammation was reduced compared to the placebo control (*p* < 7 × 10^−8^) with a SCORAD of 12.5 ± 6.6 after 8 weeks. • Displayed (*p* < 0.05) wound healing activity in mice with a 50% effective dosage (ED_50_) of 155 µg/g compared to the control.	(180–182)
Apigenin	*Matricaria recutita* L.	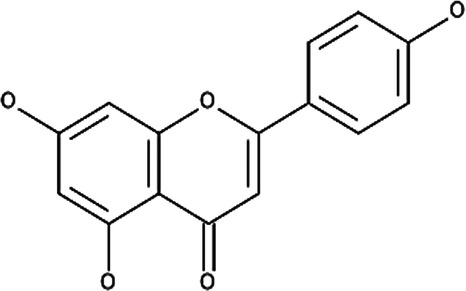	Cyclooxygenase 2 (COX-2) synthesis, interleukin 4 (IL-4) and IL-13	• Reduced the expression of 12-O-tetradecanoylphorbol-13-acetate (TPA) induced COX-2 on human leukemia (SKH-1) cells at a concentration of 20 µmols (below 1.0 × 10^5^ pg PGE2/mg of protein, *p* < 0.05) compared to the TPA vehicle control (around 2.0 × 10^5^ pg PGE2/mg of protein). • Inhibited IL-4 synthesis with a 50% inhibitory concentration (IC_50_) of 5.7 µM against anti-immunoglobulin E (IgE) antibody stimulated basophils and 10.2 µM against anti-CD3 antibody stimulated peripheral blood mononuclear cells (PBMCs) at 30 µM. • Inhibited IL-13 synthesis by 90% at 30 µM.	(183, 184)
Caffeic acid	*Calendula officinalis* L	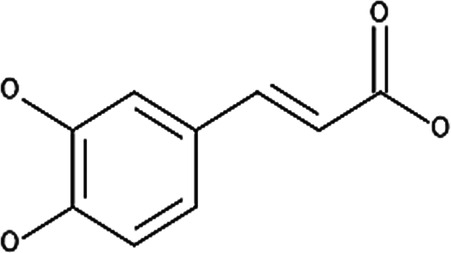	Inhibits Poly-p-methoxyphenethylmethylamine (Compound 48/80), which induces scratching behavior and reduces histamine content	• Inhibited the scratching behavior induced by compound 48/80 after 2 weeks of exposure (*p* < 0.05) to just above 40 scratches per hour at 200 mg/kg in comparison to 90 scratches per hour when exposed to the ICR or albino outbred mouse strain control. • Improved the histamine changes caused by compound 48/80 in the wet tissue from 10.7 ± 0.6 to 14.0 ± 0.8 µg/g (*p* < 0.05) at 200 mg/kg.	(185)
Luteolin	*Matricaria recutita* L.	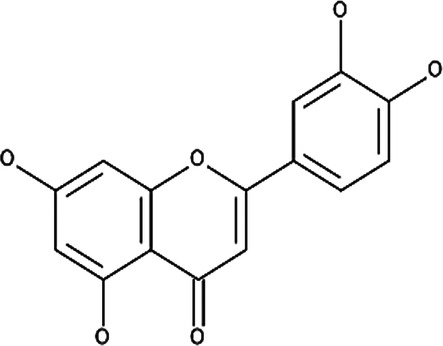	IL-4 and IL-13	• Inhibited IL-4 synthesis with an IC_50_ of 2.4 µM against anti-IgE antibodies stimulated basophils and 10.2 µM against anti-CD3 antibody stimulated PBMCs at 30 µM. • Inhibited IL-13 synthesis by 90% at 30 µM.	(184)
Sesquiterpene lactones (helenalin)	*Arnica montana* L.	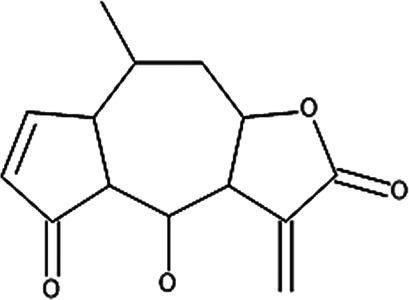	Nuclear factor kappa beta (NF-κB), IL-1, IL-8 and tumor necrosis factor alpha (TNF-α)	• Reduced the expression of NF-κB p65 protein in human ovarian cancer cells (A2780) at 2 µM. • Reduced IL-1β (*p* < 0.01) from approximately 14 to between 11 and 12 pg/mL, IL-8 from approximately 85–45 pg/mL and TNF-α (*p* < 0.0001) from approximately 14–18 pg/mL at 0.02 µM when compared to lipopolysaccharide (LPS) simulated human keratinocyte (HaCaT) cells	(186, 187)

The oil extracted from the flowers should be a minimum of 4 mL/kg of raw material as α-bisabolol and other sesquiterpenes and terpenoids have been isolated from the oils, which have been shown to possess anti-allergic, anti-inflammatory and antispasmodic properties (126). Adverse effects experienced when applying the treatment may be due to apigenin which is able to inhibit 5-lipoxygenase (5-LOX), which in turn reduces the proliferation of the epidermis potentially leading to an impaired development of the skin barrier (128, 129, 131, 132).

### 7.3 *Arnica montana* L.

*Arnica montana* L. is an herbaceous perennial plant that is located at high altitudes in the mountains and alpine meadows of Europe's continental regions. Commonly known as wolf's bane, *A. montana* has been used traditionally to treat various conditions and ailments. Ethanolic and fresh tinctures from the flower heads are administered orally while creams, ointments and wet poultices of various parts of the plant are applied topically due to their antibacterial, anti-inflammatory, immunomodulatory, anti-rheumatic and wound healing properties (133, 134).

Various constituents have been isolated from *A. montana* including sesquiterpene lactones, thymol derivatives and flavonoids (134, 135). Sesquiterpene lactones, specifically helenalin, have been shown to inhibit the production of NF-κB and pro-inflammatory cytokine production including IL-1, IL-8 and TNF-α, while thymol derivatives and phenolic acids possess anti-fungal potential and are used as preservatives (Table 6) (129, 135). In light of this, *A. montana* could potentially be used to alleviate symptoms associated with nummular eczema and atopic dermatitis, however, clinical trials in humans are required to evaluate the efficacy of the treatment.

Due to the plant's vast medicinal potential, *A. montana* was overharvested in the wild resulting in the plant being placed under the European Union Habitats Directive and the European Union regulation of trade of fauna and flora restricting the harvesting of wild species. Attempts to cultivate *A. montana* were conducted and a clone with high yield was cultivated in the 1990s, which is used in most *A. montana-*based products (133, 136). Other factors associated with cultivation, including the planting date, fertilizer type and mode of reproduction were considered by Pljevljakušić et al. to improve the standardization of *A. montana* (137).

### 7.4 *Calendula officinalis* L.

Similar to *A. montana*, *Calendula officinalis* L. has a long history of traditional use and has been used in various treatments. This herb, commonly referred to as marigold, is indigenous to Europe but due to its popularity as an ornamental plant, it can be found worldwide. Traditionally, tinctures prepared from the flowers have been used to reduce swelling, while the sap has been used to lower body temperatures and treat pain (138). With this in mind, various herbal-based formulations have been prepared using the flowers to relieve ear pain and oedema associated with acute musculoskeletal injuries (139). To lower cholesterol levels and reduce blood pressure the flowers are boiled in water and consumed (138).

Various chemical constituents have been isolated from *C. officinalis* including terpenoids, flavonoids, phenolic acid, carotenoids and steroids. Some of the most common compounds isolated include rutin, caffeic acid, chlorogenic acid and isorhamnetin-3-glycoside (128, 140). These constituents contribute to *C. officinalis* extensive pharmacological effects, which include antimicrobial, anti-helminthic, anti-inflammatory, antioxidant and hepatoprotective properties (Table 6) (139). *Calendula officinalis* has previously displayed anti-inflammatory properties, but studies have indicated that the plant may impair the development of the skin barrier as isorhamnetin-3-glycosides inhibits the production of lipoxygenase (128, 131, 132). Therefore, caution should be considered when using this treatment as a natural alternative to non-steroid-based treatments, which assist with alleviating the itching sensation associated with eczema.

## 8 Discussion

This review focused on discussing the potential etiology and pathogenesis of various types of eczema including atopic dermatitis, contact dermatitis, dyshidrotic eczema, asteatotic dermatitis, nummular eczema and seborrheic dermatitis with an overview on the socioeconomic impact in developing countries like South Africa. This review also covered conventional, targeted and alternative treatments that are currently on the market.

Patients with contact dermatitis are often unaware of which allergens trigger their symptoms, consequently in 2013, a clinical evaluation was conducted in Northern Ethiopia to identify allergens which caused ACD in both urban and rural communities. A patch test was conducted on 480 volunteers who were suspected of having ACD. Each patient was tested against 19 known allergens where nickel sulphate (26.2%), p-tert-butylphenol formaldehyde resin (10%), fragrance mix (7.1%), potassium dichromate (5.4%) and cobalt chloride (4.6%) displayed the most positive patch test reactions. These five allergens are closely linked to occupational tasks commonly performed by hairdressers, construction workers, cleaners, healthcare personnel, and metal industry professionals. This suggests a strong association between ACD and everyday work-related activities (141).

In most studies that utilize patch tests to identify potential allergens, lighter skin types are used. This is because symptoms including erythema and vesicles, which are used to detect early stages of ACD, are not easily visible in darker skin types. In some cases, early onset of ACD in darker skin types has been linked to lichenification and hyperpigmentation but is often misdiagnosed as seborrheic dermatitis if present on the scalp. These challenges lead to delays in diagnosis and affect research conducted on ACD as the results are predominantly reported on lighter skin types (142). Research on ACD on darker skin types should be conducted so that more effective detection methods can be developed for these skin types to facilitate diagnosis. Due to the link between endocannabinoids and ACD, alternative treatments such as 2-arachidonoylglycerol (2-AG), anandamide (AEA), *Δ* (9)-tetrahydrocannabinol (THC), cannabidiol (CBD) and palmitoylethanolamine (PEA) should be investigated for their effect on ACD. These cannabinoid treatments either promote the activation of CB1 and CB2, while reducing the activation of TRPV1 as seen with 2-AG, AEA and THC, or target TPRV1, as with CBD and PEA (45).

As mentioned in Section 4.1, prevalence rates of 35.3% and 30.8% were reported among different age groups in 2009 across Africa, however, information on the socioeconomic impact of eczema is limited within Africa and South Africa. To help bridge this gap, Table 2 includes estimated costs for dyshidrotic dermatitis, adjusted to reflect 2025 economic values. These figures are estimated costs and therefore, additional studies regarding the overall economic burden of various types of eczema and treatments available within South Africa and across the African may need to be considered to provide a more comprehensive understanding on the condition's socioeconomic impact.

The predominantly used conventional treatment is corticosteroids, however, corticosteroids are known for their cutaneous and systemic adverse effects, which are potentially enhanced when corticosteroids are used over extended periods or high-potency levels are administered. The most common cutaneous adverse effects include atrophy, rosacea, secondary infections and contact dermatitis. Systemic adverse effects include cataracts, hypertension and hyperglycemia, which can be minimized if low-potency treatments are used (83). Another common symptom associated with the extended use of corticosteroids is tachyphylaxis (acute tolerance) where the effectiveness of the treatment begins to diminish over time. There is speculation as to whether patients become resistant to a treatment as studies supporting these claim are underexplored (81, 83).

As a second-line treatment, phototherapy has been recommended for most types of eczema with varying effects. In some cases, phototherapy initiates an allergic reaction causing a condition known as photoallergic contact dermatitis (PACD), which occurs when patients, previously exposed to photosensitization agents, are exposed to UVA or visible light (400–700 nm) initiating a T-cell-mediated hypersensitivity reaction (143). In most cases, PACD is considered uncommon, however, a review conducted in Canada evaluated cases of PACD between January 2001 and December 2010 and found that 33.8% patients suffered from PACD indicating that the condition is not as uncommon as previously perceived (144).

A study conducted by Polderman et al. found that UVA-1 and PUVA displayed similar effects against dyshidrotic dermatitis, however, the authors mentioned that PUVA may increase a patient's risk of developing cancer. The authors focused on UVA-1 and found that the affected areas were significantly reduced (*p* < 0.039) compared to the placebo after three weeks of treatment (145). Polderman et al. speculation on the carcinogenic potential of PUVA supported the views of Lindelof and Sigurgeirsson who evaluated the correlation of various cancers in conjunction with the use of PUVA between 1974 and 1985 in Sweden. The authors found that the occurrence of cutaneous squamous carcinoma increased six-fold in male patients, while an increase of five-fold was observed in female patients. Furthermore, both sexes were at a significant risk (*p* < 0.05) of developing respiratory cancers. Other cancers that were correlated with the use of PUVA include colon, kidney and pancreatic cancer (146).

Ahad et al. evaluated the development of skin cancer in 925 eczema patients between 1996 and 2018 that were treated with BBUVB, NBUVB or UVA and BBUVB concurrently, and found that 14 patients had suffered from one of the main types of skin cancer specifically melanoma, squamous cell carcinoma and basal cell carcinoma. The authors concluded that the number of patients with their first occurrence of skin cancer and the total number of new cases after treatments, based on the patient-based age-standardized incidence rate (ASIR), were 137 per 100,000 person-years and 256 per 100,000 person-years, respectively indicating that there was a low skin cancer incident rate amongst these patients (147). Considering both Ahad et al. and Polderman et al. results, phytotherapy is an effective treatment, however, caution should be advised when using UVB as this ray has the potential to cause various skin cancers. Using UVA and PUVA to treat eczema should be reduced to less than three weeks as the risk of cancer is considerably higher due to the ray's ability to penetrate the dermal layer.

If corticosteroids and UV treatments are no longer effective, immunosuppressive agents such as azathioprine and mycophenolate are used. The mechanism of action for azathioprine is under-researched, however, a study conducted by Halliday et al. evaluated the effect of azathioprine on adenosine triphosphatase (ATPase) positive Langerhans cells within a mouse epidermis model. The authors found that azathioprine significantly reduced the number of epidermal Langerhans cells by 51% (*p* < 0.01) and blood leukocytes by 42.84% (*p* < 0.05) (86). Patients with atopic and contact dermatitis have previously been shown to have a higher count of Langerhans cells. Hussein obtained 20 eczematous tissues from the Department of Pathology and evaluated the number of Langerhans cells present in the epidermis and dermis compared to 20 normal skin tissues. During this study, the author found a higher count of Langerhans cells in both the epidermis (2.50 ± 0.16 per high power field, *p* < 0.05) and dermis (2.70 ± 0.15 per high power field, *p* < 0.05) compared to normal skin (1.20 ± 0.13 and 1.25 ± 0.09 per high power field) (148).

One of the main concerns when using azathioprine over a prolonged period is its mutagenic and carcinogenic effect. A study conducted by Bouwes et al. evaluated the association between various immunotherapies including azathioprine and skin cancers utilizing patient records between 1969 and 1994, which were collected from the Princess Alexandra Hospital. The initial dose of azathioprine after renal transplantation was 5 mg/kg of body weight per day, which was reduced to 3 mg/kg of body weight per day and maintained between 2 and 2.5 mg/kg of body weight per day. The authors correlated the use of azathioprine with the occurrence of skin cancer and found that patients using the treatment for 1, 11 and 20 years had an increased risk of developing skin cancer by 7 to 45 to 70% (149). If recommended to patients, a duration of less than a year should be considered to reduce the risk of developing skin cancer.

In most cases azathioprine is used for patients with atopic dermatitis, however, Levy et al. evaluated the effect of azathioprine on the synthesis of IgG and IgM and found that the treatment significantly lowered the synthesis of both immunoglobulins by 33.4 and 40.9%, respectively after four months of use (150). This could potentially reduce symptoms associated with seborrheic dermatitis as a study conducted by Bergbrant et al. indicated that the condition may be due to an immunological effect, reporting that of the 30 patients with seborrheic dermatitis, 14 had higher serum levels of IgA, while 11 patients had higher IgG antibody levels, and approximately 46% of patients expressed high levels of natural killer cells (66).

In 1996, Neuber et al. conducted a similar study to Bergbrant et al., while considering whether the overexposure to *Malassezia* species would affect the production of IL-2, IL-10 and IFN-γ and various immunoglobulins. The authors found that patients with seborrheic dermatitis displayed higher levels of IgA and IgG regardless of whether they were overexposed to *Malassezia* species or not. In contrast, IL-2 and IFN-γ levels were downregulated, while IL-10 was upregulated. The authors concluded that seborrheic dermatitis could be due to the overexpression of IL-10, which downregulated the production of IL-2 and IFN-γ and promoted the synthesis of IgA and IgG (151).

In contrast, one of mycophenolate mechanisms of action is to decrease the production of IL-10 and promote the expression of IFN-γ as demonstrated in a study conducted by Neuber et al. who found that after 12 weeks IFN-γ significantly increased from 2.40 to 3.00 pg/mL (*p* < 0.02), while IL-10 decreased by 65.5% (*p* < 0.001) (88). When IFN-γ is expressed, it reduces the production of IgE by inhibiting the expression of IL-4. In a study conducted by Vercelli et al., the authors found that IL-4 significantly increased the synthesis of IgE and inhibited IFN-γ between 50% and 90% (152). Mycophenolate may indirectly reduce the production of IgE and could potentially be used to treat other types of eczema including contact dermatitis. Pickenäcker et al. reported a case where mycophenolate was used to treat a patient with dyshidrotic dermatitis and found that after four weeks of treatment, symptoms associated with the condition significantly reduced, however no mechanistic studies were conducted (153).

Unlike azathioprine and mycophenolate, patients that use ciclosporin A are often monitored due to risks of various adverse effects including increase in creatinine, hypertension, gastrointestinal symptoms, headaches and paranesthesia. A study conducted by Schmitt et al. found that the use of ciclosporin increased creatinine by more than 30%, hypertension between 5.8% and 12.4% and gastrointestinal symptoms by 40.3% (92).

Recently, targeted therapies, including biologics and small molecules, have been approved for use on moderate to severe atopic dermatitis patients when conventional treatments are no longer effective. The most common biologic, dupilumab, as of 2023, was approved for use on children from 6 months of age due to its eczema area severity index (EASI), which was measured to be between 64% and 69%. In most cases patients did not experience adverse effects. Among those who did, reported issues included upper respiratory tract infections, worsening of the condition, headaches and reactions at the injection site (154, 155). Common adverse effects associated with lebrikizumab and tralokinumab include injection site reactions, erythema, conjunctivitis and nasopharyngitis (156, 157).

Nemolizumab is used only in patients 12 years of age and older. Several adverse effects have been observed including mild urticaria, hypersensitivity at the injection sites and eosinophilia (158, 159). Adverse effects associated with rocatinlimab and rademikibart were found to be mild to moderate with most patients reporting fever and chills, which is considered a common reaction when using biologics. Other adverse effects detected included headaches, reactions at the injection site and erythema (160, 161).

Compared to biologics, small molecules such as delgocitinib and ruxolitinib, have reported to be well-tolerated by atopic dermatitis patients with few reporting adverse effects such as headaches and nasopharyngitis (107). In contrast, though gusacitinib and upadacitinib are effective in reducing atopic dermatitis and dyshidrotic dermatitis, both displayed notable adverse effects including upper respiratory infections while gusacitinib has been shown to cause headaches, nausea and gastrointestinal (107, 112). Common adverse effects associated with abrocitinib include nausea, nasopharyngitis, headache, acne, diarrhea and urinary tract infections (115).

A study conducted by Bieber et al. demonstrated that patients administered with 2 and 4 mg of baricitinib displayed mild to moderate adverse effects after 16 weeks including upper respiratory tract infections and an increased risk of infection caused by the herpes simplex virus (162). Adverse effects associated with tofacitinib when administered orally include nasopharyngitis and a heightened risk of deep vein thrombosis and pulmonary embolism (107).These treatments are more effective than conventional treatments, however, they are often expensive and are considered unattainable for most households.

Due to costs, availability and the adverse effects associated with the abovementioned conventional and targeted treatments, most patients prefer to use plant based alternative methods, however, the efficacy and safety of these alternative treatments is not always well documented. As mentioned in Section 7 and Table 6, most of the information available for alternative treatments is either evidence-based or conducted on animals. Moreover, as mentioned in Section 7.1, alternative treatments may display significant effects when used on mice, however, the same effect may not be observed when evaluated on patients even though practitioners use this treatment. This limitation in efficacy testing on eczema patients' needs to be considered when evaluating alternative based treatments before they are placed on the market.

A bias associated with the safety of alternative treatments is that they are inherently safe since they are derived from plants. This may not always be the case as demonstrated by Li et al. who evaluated the effect each plant and their combination on embryotoxicity and potential skin irritation. The authors found that of all the plants used in PentaHerbs, only *Phellodendron amurense* (also known as *Phellodendron chinense* C.K.Schneid) displayed cytotoxic potential against mouse embryonic stem cells (D3) with an IC_50_ of 3.12 µg/mL and a 50% differentiation concentration (ID_50_) of 1.37 µg/mL, indicating that the plant induced embryotoxicity (163). Effects on D3 cells was attributed to the plant's secondary metabolites, specifically berberine, which have previously been shown to increase the risk of neonatal jaundice and kernicterus potentially leading to embryotoxicity (164).

The concern regarding the safety profile of *Arnica montana* and *Calendula officinalis* specifically as eczema-orientated treatments has been increasing over the years as a few studies have deduced that these plants may cause ACD as demonstrated by Reider et al. who found that patients suffer from contact dermatitis when exposed to these plants (165). This correlates to some of the active compounds isolated from these plants. As mentioned in Table 6, helenalin has previously displayed significant activity against NF-κB, IL-1β, IL-8 and TNF-α, however, the consumption of tinctures containing *A. montana* has declined as helenalin is considered a toxin causing skin irritation, severe gastroenteritis and internal bleeding of the digestive tract (135).

*Calendula officinalis* was found to impair the development of the skin barrier as it promoted TEWL indicating that exposure to *C. officinalis* could worsen eczema related symptoms as the severity of the condition is linked to the magnitude of the skin barrier's dysfunction (166, 167). Unlike *A. montana* and *C. officinalis*, the safety profile of German chamomile has not been considered, however, studies have been conducted on the secondary metabolites present in the plant. Paulsen et al. evaluated the effect of herniarin as a possible allergen. The authors recruited 36 patients and found that four of these patients experienced contact dermatitis when exposed to herniarin (168).

Jacob et al. evaluated the effect of bisabolol on seven patients and found that four experienced an allergic reaction when exposed to the constituent (169). Therefore, if plant-based products are potentially effective in reducing symptoms associated with eczema, precautions should be taken and *in vivo* based studies should be conducted to ensure the secondary metabolites present in the plant do not cause adverse effects when administered.

## 9 Conclusion

Extensive research has been conducted on atopic dermatitis regarding the condition's etiology, treatment and socioeconomic impact, however, limited emphasis has been placed on the other types of eczema. This review aimed to collate information on all identified types of eczema as well as their potential etiology and pathogenesis, however, information on dyshidrotic eczema, asteatotic dermatitis and nummular dermatitis remains insufficient. The socioeconomic impact of eczema in South Africa should be considered as few accredited sources are currently available on how the condition affects the economy. There is also a lack of recent research on eczema, the various types and its socioeconomic impact as many of the publications used in this study were published before the year 2020.

This review focused on various treatments available including conventional, targeted and alternative treatments. Conventional treatments remain the most widely used, however there are various concerns regarding their adverse effects, including reports on the potential carcinogenicity of phototherapies and immunosuppressors. Although conventional treatments are often used for any type of eczema, their direct efficacy in other types of eczema, other than atopic dermatitis, remains under investigation. Further research should therefore focus on the direct effectiveness of these conventional treatments against each type of eczema. The most effective alternative to conventional treatments is targeted therapies, which have been FDA approved and clinically evaluated. Unfortunately, these treatments are only available in certain countries, are costly and can only be used by those with moderate to severe symptoms. Moreover, the effect of targeted therapies on other types of eczema is currently under-researched and should be considered. Existing reports on the effectiveness of currently available alternative treatments, including TCM and natural Western treatments, to treat eczema remain sparse.

In conclusion, more current research on the various types of eczema's etiology and pathogenesis should be considered as well as their socioeconomic effect on developing countries. Practitioners should inform consumers about the benefits and potential risks of both conventional and alternative treatments as many cause severe adverse effects when used over a prolonged period or may contain toxic properties. Further research into effective conventional and alternative medicine that has fewer adverse effects and can be used for prolonged periods of time should be considered.
